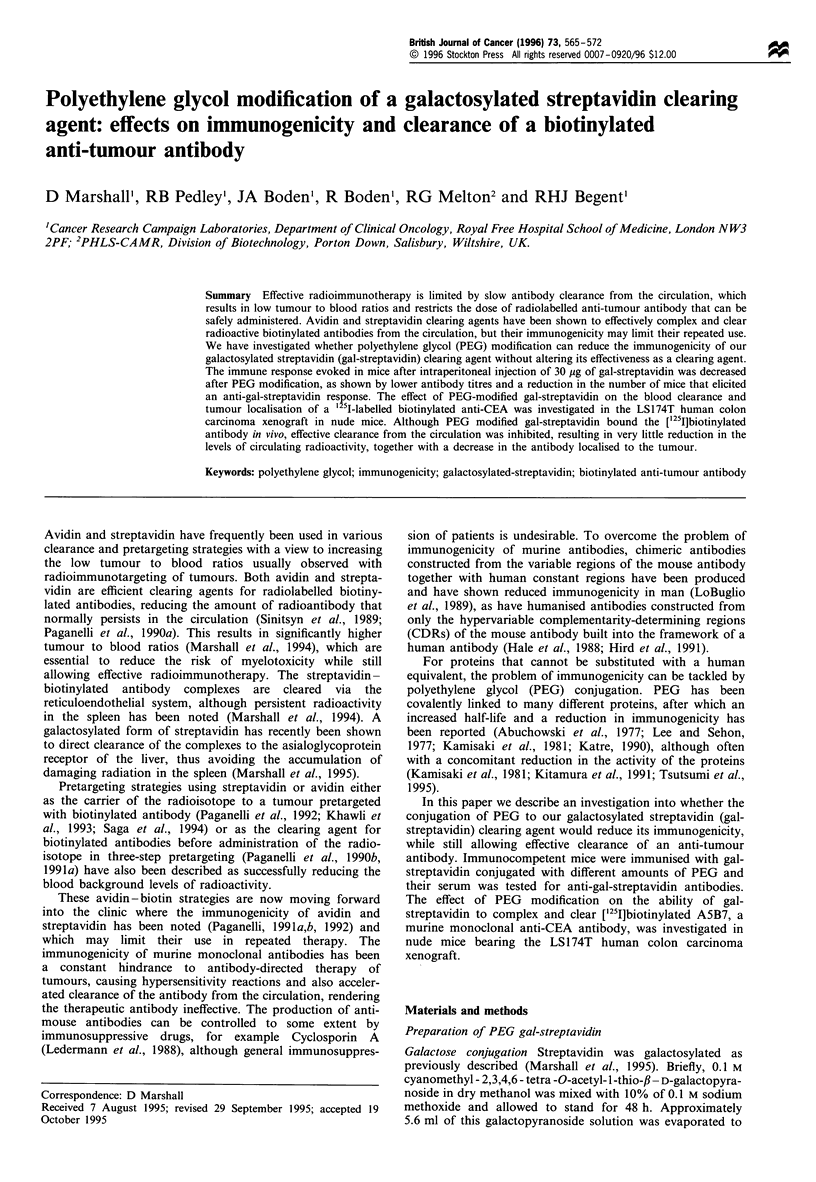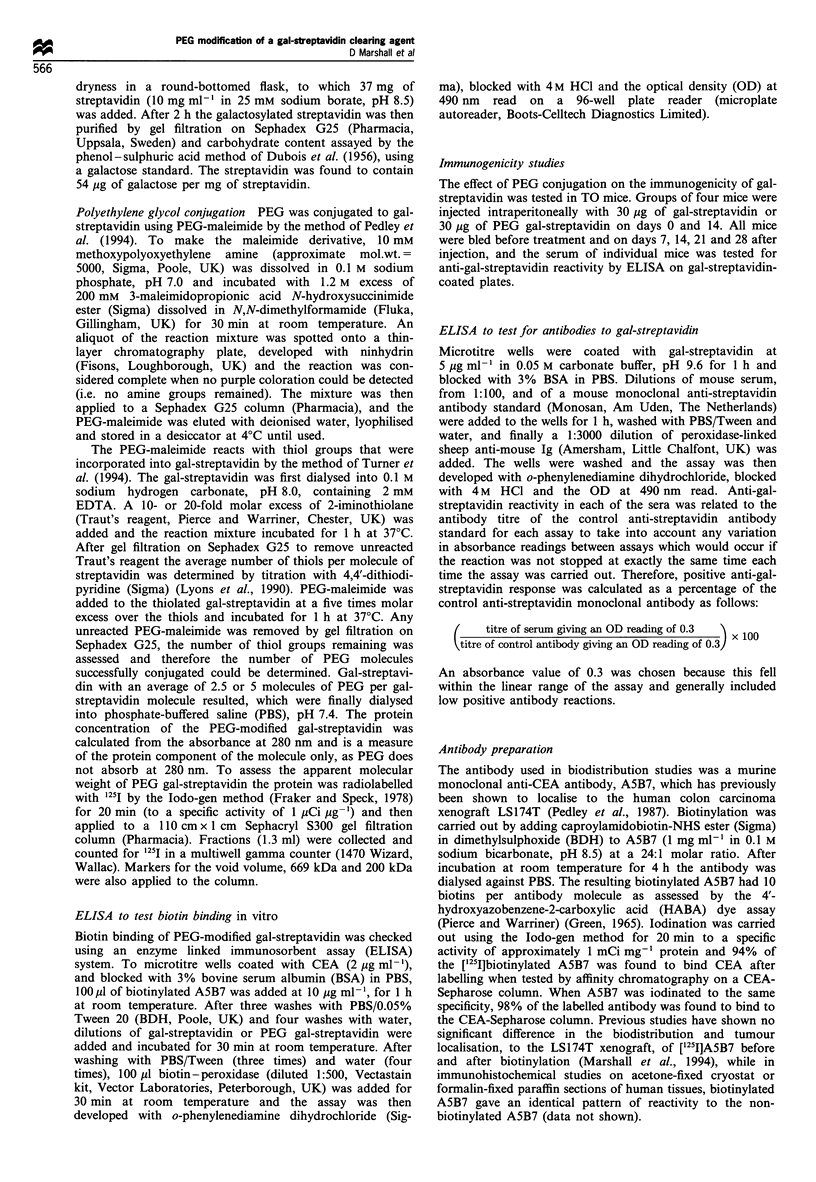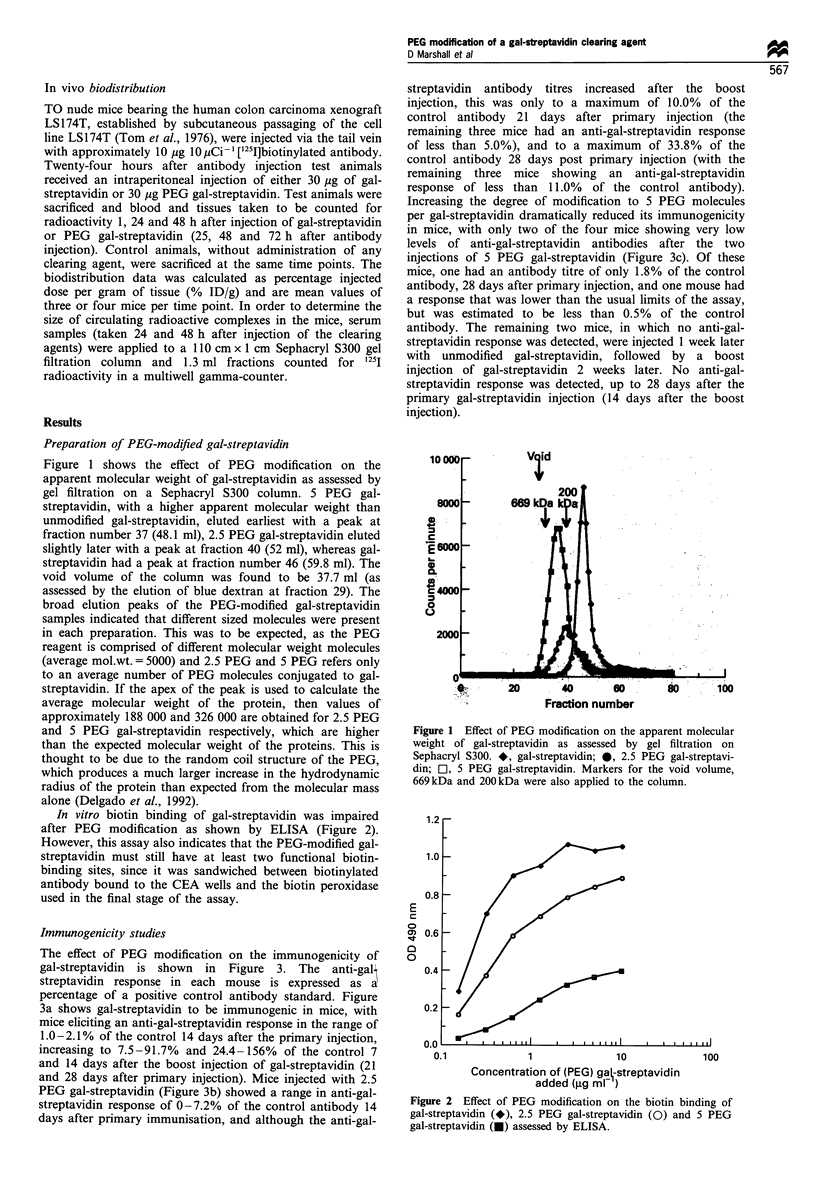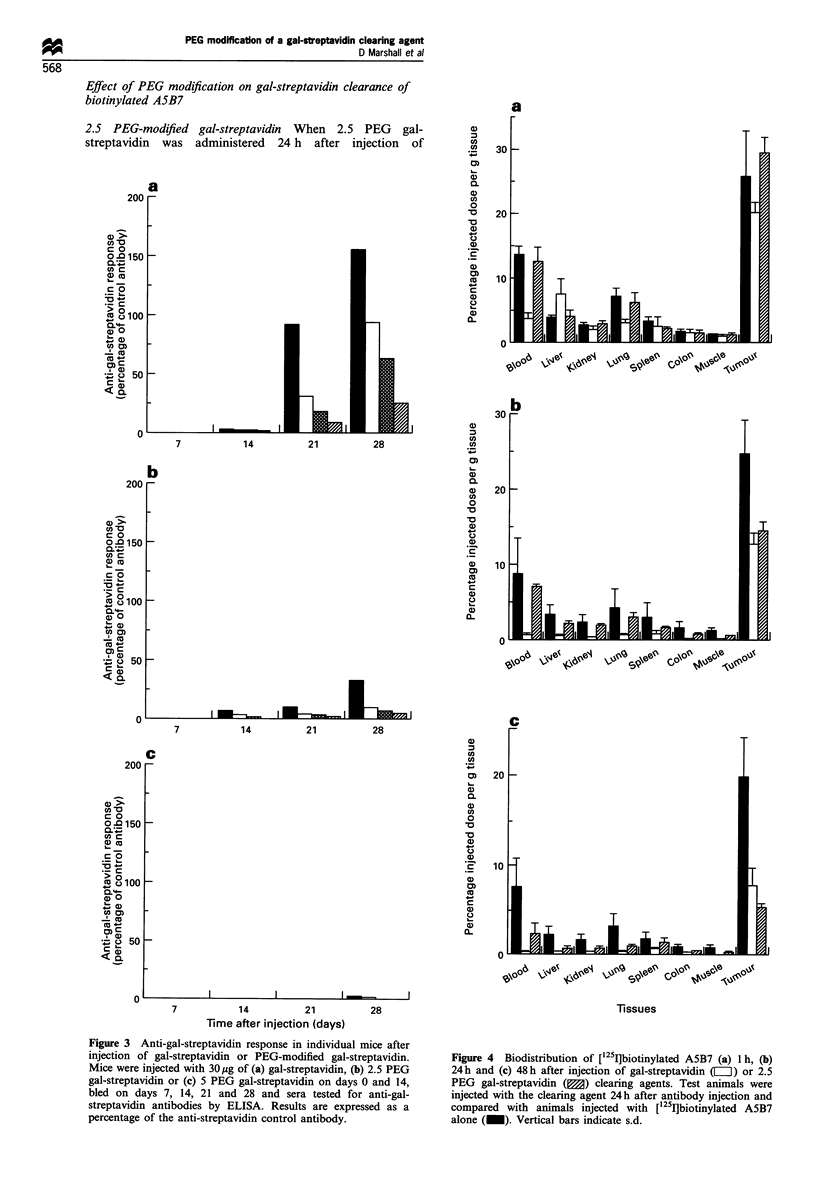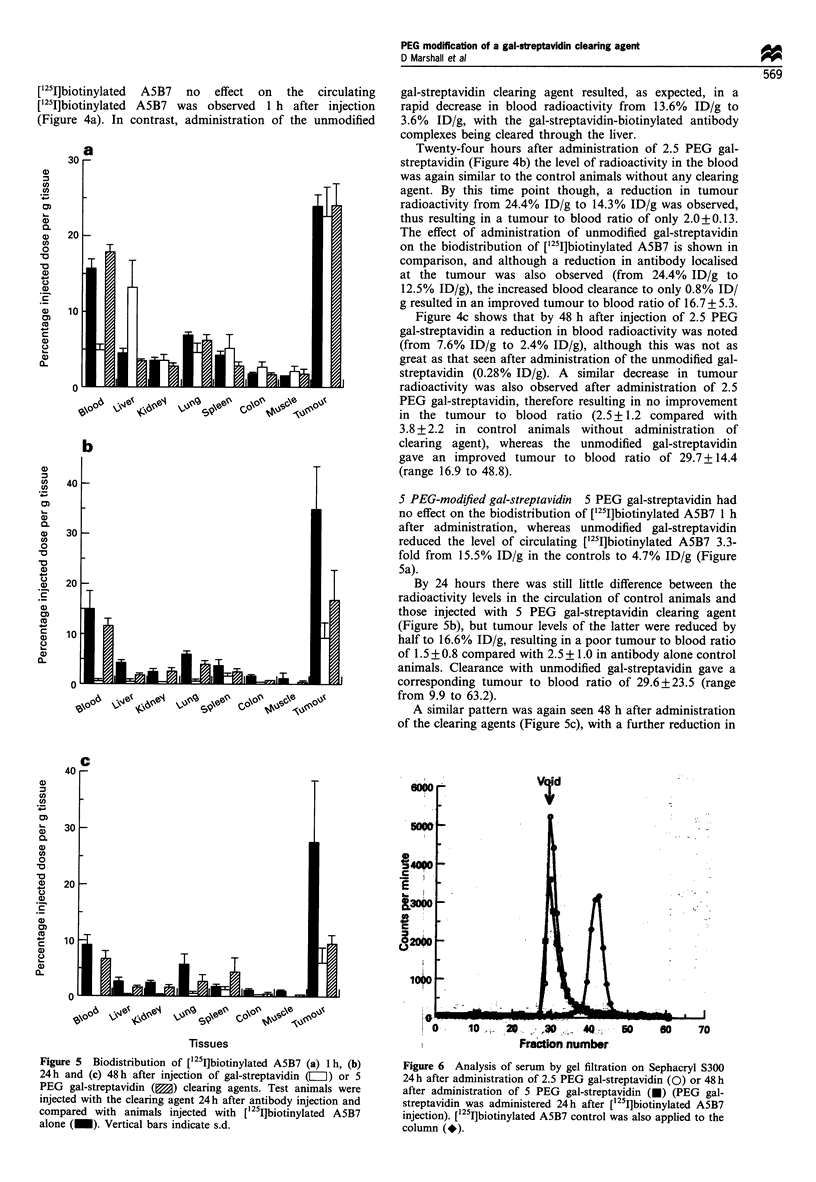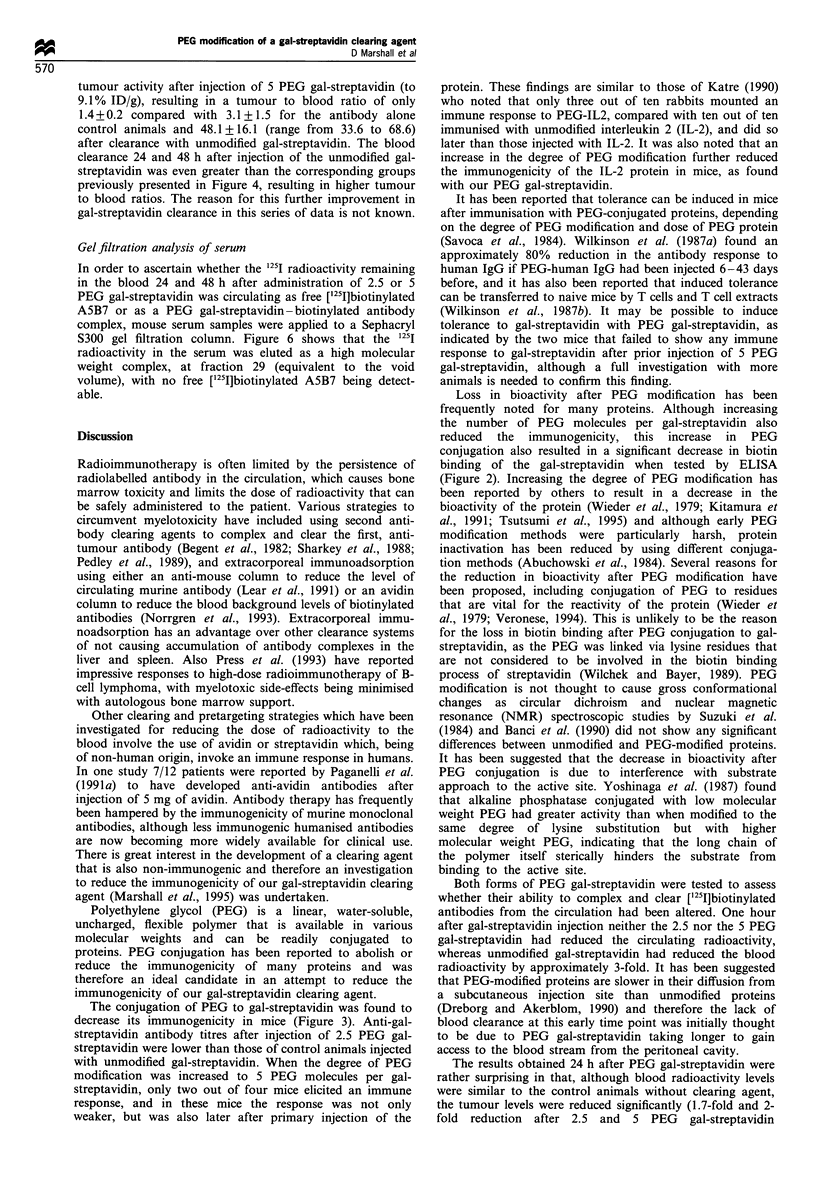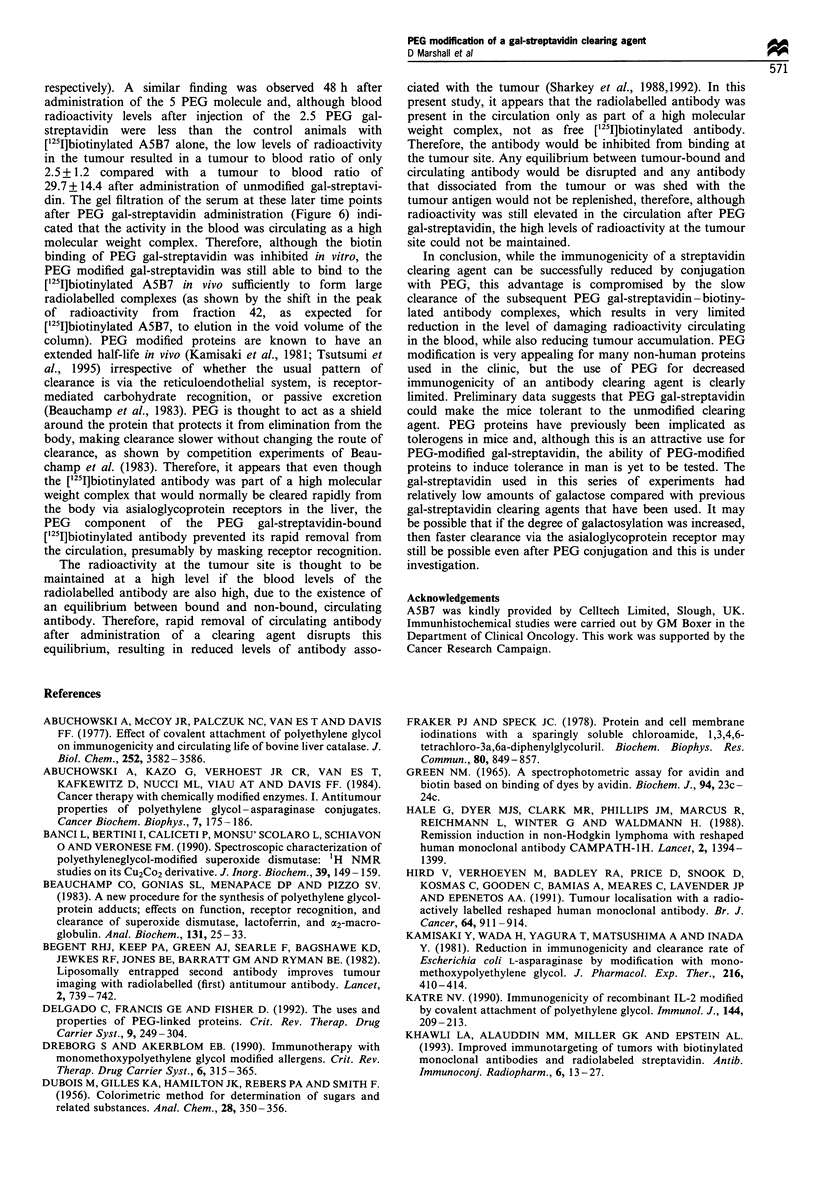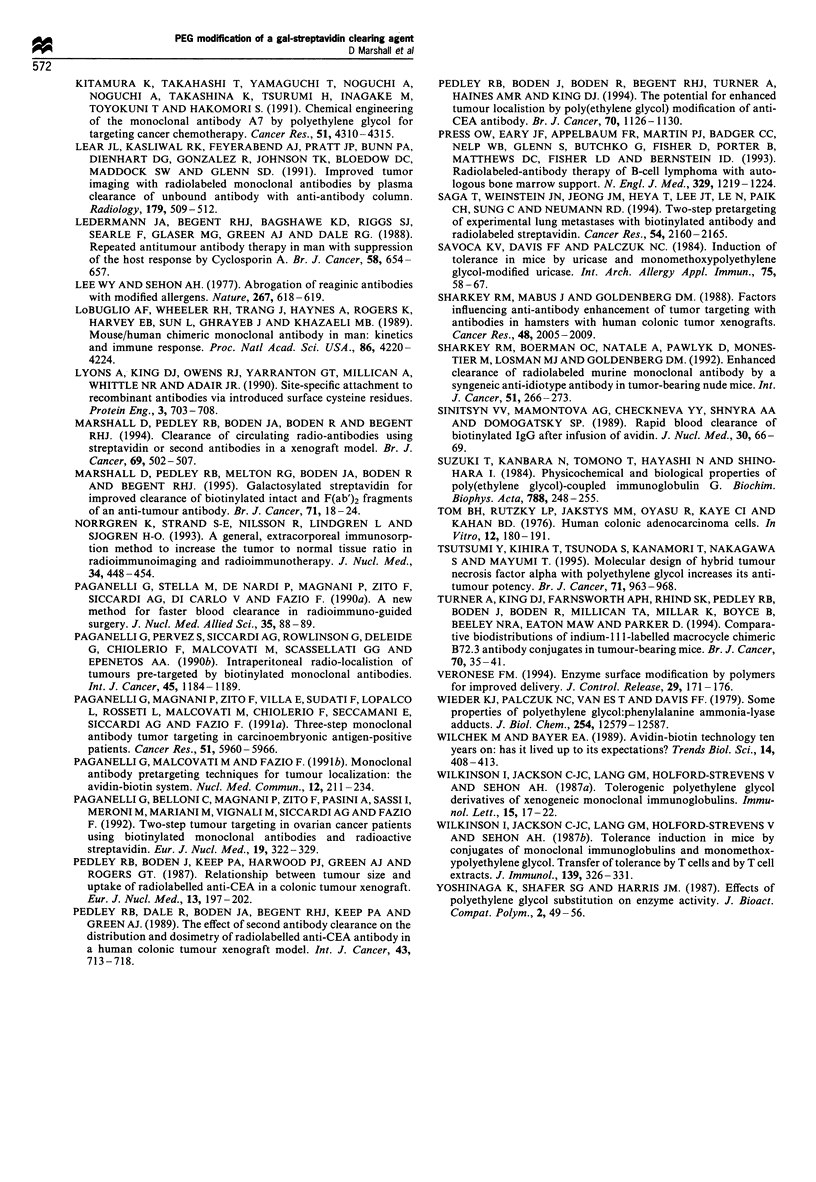# Polyethylene glycol modification of a galactosylated streptavidin clearing agent: effects on immunogenicity and clearance of a biotinylated anti-tumour antibody.

**DOI:** 10.1038/bjc.1996.99

**Published:** 1996-03

**Authors:** D. Marshall, R. B. Pedley, J. A. Boden, R. Boden, R. G. Melton, R. H. Begent

**Affiliations:** Cancer Research Campaign Laboratories, Department of Clinical Oncology, Royal Free Hospital School of Medicine, London, UK.

## Abstract

Effective radioimmunotherapy is limited by slow antibody clearance from the circulation, which results in low tumour to blood ratios and restricts the dose of radiolabelled anti-tumour antibody that can be safely administrated. Avidin and streptavidin clearing agents have been shown to effectively complex and clear radioactive biotinylated antibodies from the circulation, but their immunogenicity may limit their repeated use. We have investigated whether polyethylene glycol (PEG) modification can reduce the immunogenicity of our galactosylated streptavidin (gal-streptavidin) clearing agent without altering its effectiveness as a clearing agent. The immune response evoked in mice after intraperitoneal infection of 30 micrograms of gal-streptavidin was decreased after PEG modification, as shown by lower antibody titres and a reduction in the number of mice that elicited an anti-gal-streptavidin response. The effect of PEG-modified gal-streptavidin on the blood clearance and tumour localisation of a 125I-labelled biotinylated anti-CEA was investigated in the LS174T human colon carcinoma xenograft in nude mice. Although PEG modified gal-streptavidin bound the [125I]biotinylated antibody in vivo, effective clearance from the circulation was inhibited, resulting in very little reduction in the levels of circulation radioactivity, together with a decrease in the antibody localised to the tumour.


British Journal of Cancer (1996) 73, 565-572
© 1996 Stockton Press All rights reserved 0007-0920/96 $12.00

### Polyethylene glycol modification of a galactosylated streptavidin clearing agent: effects on immunogenicity and clearance of a biotinylated anti-tumour antibody

D Marshall[1], RB Pedley[1], JA Boden[1], R Boden[1], RG Melton[2] and RHJ Begent[1]

[1]*Cancer Research Campaign Laboratories, Department of Clinical Oncology, Royal Free Hospital School of Medicine, London NW3 2PF;* [2]*PHLS-CAMR, Division of Biotechnology, Porton Down, Salisbury, Wiltshire, UK.*

**Summary** Effective radioimmunotherapy is limited by slow antibody clearance from the circulation, which results in low tumour to blood ratios and restricts the dose of radiolabelled anti-tumour antibody that can be safely administered. Avidin and streptavidin clearing agents have been shown to effectively complex and clear radioactive biotinylated antibodies from the circulation, but their immunogenicity may limit their repeated use. We have investigated whether polyethylene glycol (PEG) modification can reduce the immunogenicity of our galactosylated streptavidin (gal-streptavidin) clearing agent without altering its effectiveness as a clearing agent. The immune response evoked in mice after intraperitoneal injection of 30 $\mu$g of gal-streptavidin was decreased after PEG modification, as shown by lower antibody titres and a reduction in the number of mice that elicited an anti-gal-streptavidin response. The effect of PEG-modified gal-streptavidin on the blood clearance and tumour localisation of a $^{125}$I-labelled biotinylated anti-CEA was investigated in the LS174T human colon carcinoma xenograft in nude mice. Although PEG modified gal-streptavidin bound the [$^{125}$I]biotinylated antibody *in vivo*, effective clearance from the circulation was inhibited, resulting in very little reduction in the levels of circulating radioactivity, together with a decrease in the antibody localised to the tumour.

**Keywords:** polyethylene glycol; immunogenicity; galactosylated-streptavidin; biotinylated anti-tumour antibody

Avidin and streptavidin have frequently been used in various clearance and pretargeting strategies with a view to increasing the low tumour to blood ratios usually observed with radioimmunotargeting of tumours. Both avidin and streptavidin are efficient clearing agents for radiolabelled biotinylated antibodies, reducing the amount of radioantibody that normally persists in the circulation (Sinitsyn *et al.*, 1989; Paganelli *et al.*, 1990*a*). This results in significantly higher tumour to blood ratios (Marshall *et al.*, 1994), which are essential to reduce the risk of myelotoxicity while still allowing effective radioimmunotherapy. The streptavidin–biotinylated antibody complexes are cleared via the reticuloendothelial system, although persistent radioactivity in the spleen has been noted (Marshall *et al.*, 1994). A galactosylated form of streptavidin has recently been shown to direct clearance of the complexes to the asialoglycoprotein receptor of the liver, thus avoiding the accumulation of damaging radiation in the spleen (Marshall *et al.*, 1995).

Pretargeting strategies using streptavidin or avidin either as the carrier of the radioisotope to a tumour pretargeted with biotinylated antibody (Paganelli *et al.*, 1992; Khawli *et al.*, 1993; Saga *et al.*, 1994) or as the clearing agent for biotinylated antibodies before administration of the radioisotope in three-step pretargeting (Paganelli *et al.*, 1990*b*, 1991*a*) have also been described as successfully reducing the blood background levels of radioactivity.

These avidin–biotin strategies are now moving forward into the clinic where the immunogenicity of avidin and streptavidin has been noted (Paganelli, 1991*a*,*b*, 1992) and which may limit their use in repeated therapy. The immunogenicity of murine monoclonal antibodies has been a constant hindrance to antibody-directed therapy of tumours, causing hypersensitivity reactions and also accelerated clearance of the antibody from the circulation, rendering the therapeutic antibody ineffective. The production of anti-mouse antibodies can be controlled to some extent by immunosuppressive drugs, for example Cyclosporin A (Ledermann *et al.*, 1988), although general immunosuppression of patients is undesirable. To overcome the problem of immunogenicity of murine antibodies, chimeric antibodies constructed from the variable regions of the mouse antibody together with human constant regions have been produced and have shown reduced immunogenicity in man (LoBuglio *et al.*, 1989), as have humanised antibodies constructed from only the hypervariable complementarity-determining regions (CDRs) of the mouse antibody built into the framework of a human antibody (Hale *et al.*, 1988; Hird *et al.*, 1991).

For proteins that cannot be substituted with a human equivalent, the problem of immunogenicity can be tackled by polyethylene glycol (PEG) conjugation. PEG has been covalently linked to many different proteins, after which an increased half-life and a reduction in immunogenicity has been reported (Abuchowski *et al.*, 1977; Lee and Sehon, 1977; Kamisaki *et al.*, 1981; Katre, 1990), although often with a concomitant reduction in the activity of the proteins (Kamisaki *et al.*, 1981; Kitamura *et al.*, 1991; Tsutsumi *et al.*, 1995).

In this paper we describe an investigation into whether the conjugation of PEG to our galactosylated streptavidin (gal-streptavidin) clearing agent would reduce its immunogenicity, while still allowing effective clearance of an anti-tumour antibody. Immunocompetent mice were immunised with gal-streptavidin conjugated with different amounts of PEG and their serum was tested for anti-gal-streptavidin antibodies. The effect of PEG modification on the ability of gal-streptavidin to complex and clear [$^{125}$I]biotinylated A5B7, a murine monoclonal anti-CEA antibody, was investigated in nude mice bearing the LS174T human colon carcinoma xenograft.

### Materials and methods

### *Preparation of PEG gal-streptavidin*

*Galactose conjugation* Streptavidin was galactosylated as previously described (Marshall *et al.*, 1995). Briefly, 0.1 M cyanomethyl-2,3,4,6-tetra-*O*-acetyl-1-thio-$\beta$-D-galactopyranoside in dry methanol was mixed with 10% of 0.1 M sodium methoxide and allowed to stand for 48 h. Approximately 5.6 ml of this galactopyranoside solution was evaporated to

Correspondence: D Marshall
Received 7 August 1995; revised 29 September 1995; accepted 19 October 1995

dryness in a round-bottomed flask, to which 37 mg of streptavidin (10 mg $ml^{-1}$ in 25 mM sodium borate, pH 8.5) was added. After 2 h the galactosylated streptavidin was then purified by gel filtration on Sephadex G25 (Pharmacia, Uppsala, Sweden) and carbohydrate content assayed by the phenol–sulphuric acid method of Dubois *et al.* (1956), using a galactose standard. The streptavidin was found to contain 54 $\mu$g of galactose per mg of streptavidin.

*Polyethylene glycol conjugation* PEG was conjugated to gal-streptavidin using PEG-maleimide by the method of Pedley *et al.* (1994). To make the maleimide derivative, 10 mM methoxypolyoxyethylene amine (approximate mol.wt. = 5000, Sigma, Poole, UK) was dissolved in 0.1 M sodium phosphate, pH 7.0 and incubated with 1.2 M excess of 200 mM 3-maleimidopropionic acid *N*-hydroxysuccinimide ester (Sigma) dissolved in *N,N*-dimethylformamide (Fluka, Gillingham, UK) for 30 min at room temperature. An aliquot of the reaction mixture was spotted onto a thin-layer chromatography plate, developed with ninhydrin (Fisons, Loughborough, UK) and the reaction was considered complete when no purple coloration could be detected (i.e. no amine groups remained). The mixture was then applied to a Sephadex G25 column (Pharmacia), and the PEG-maleimide was eluted with deionised water, lyophilised and stored in a desiccator at 4°C until used.

The PEG-maleimide reacts with thiol groups that were incorporated into gal-streptavidin by the method of Turner *et al.* (1994). The gal-streptavidin was first dialysed into 0.1 M sodium hydrogen carbonate, pH 8.0, containing 2 mM EDTA. A 10- or 20-fold molar excess of 2-iminothiolane (Traut's reagent, Pierce and Warriner, Chester, UK) was added and the reaction mixture incubated for 1 h at 37°C. After gel filtration on Sephadex G25 to remove unreacted Traut's reagent the average number of thiols per molecule of streptavidin was determined by titration with 4,4′-dithiodipyridine (Sigma) (Lyons *et al.*, 1990). PEG-maleimide was added to the thiolated gal-streptavidin at a five times molar excess over the thiols and incubated for 1 h at 37°C. Any unreacted PEG-maleimide was removed by gel filtration on Sephadex G25, the number of thiol groups remaining was assessed and therefore the number of PEG molecules successfully conjugated could be determined. Gal-streptavidin with an average of 2.5 or 5 molecules of PEG per gal-streptavidin molecule resulted, which were finally dialysed into phosphate-buffered saline (PBS), pH 7.4. The protein concentration of the PEG-modified gal-streptavidin was calculated from the absorbance at 280 nm and is a measure of the protein component of the molecule only, as PEG does not absorb at 280 nm. To assess the apparent molecular weight of PEG gal-streptavidin the protein was radiolabelled with $^{125}$I by the Iodo-gen method (Fraker and Speck, 1978) for 20 min (to a specific activity of 1 $\mu$Ci $\mu g^{-1}$) and then applied to a 110 cm × 1 cm Sephacryl S300 gel filtration column (Pharmacia). Fractions (1.3 ml) were collected and counted for $^{125}$I in a multiwell gamma counter (1470 Wizard, Wallac). Markers for the void volume, 669 kDa and 200 kDa were also applied to the column.

### *ELISA to test biotin binding* in vitro

Biotin binding of PEG-modified gal-streptavidin was checked using an enzyme linked immunosorbent assay (ELISA) system. To microtitre wells coated with CEA (2 $\mu$g $ml^{-1}$), and blocked with 3% bovine serum albumin (BSA) in PBS, 100 $\mu$l of biotinylated A5B7 was added at 10 $\mu$g $ml^{-1}$, for 1 h at room temperature. After three washes with PBS/0.05% Tween 20 (BDH, Poole, UK) and four washes with water, dilutions of gal-streptavidin or PEG gal-streptavidin were added and incubated for 30 min at room temperature. After washing with PBS/Tween (three times) and water (four times), 100 $\mu$l biotin–peroxidase (diluted 1:500, Vectastain kit, Vector Laboratories, Peterborough, UK) was added for 30 min at room temperature and the assay was then developed with *o*-phenylenediamine dihydrochloride (Sigma), blocked with 4 M HCl and the optical density (OD) at 490 nm read on a 96-well plate reader (microplate autoreader, Boots-Celltech Diagnostics Limited).

### *Immunogenicity studies*

The effect of PEG conjugation on the immunogenicity of gal-streptavidin was tested in TO mice. Groups of four mice were injected intraperitoneally with 30 $\mu$g of gal-streptavidin or 30 $\mu$g of PEG gal-streptavidin on days 0 and 14. All mice were bled before treatment and on days 7, 14, 21 and 28 after injection, and the serum of individual mice was tested for anti-gal-streptavidin reactivity by ELISA on gal-streptavidin-coated plates.

### *ELISA to test for antibodies to gal-streptavidin*

Microtitre wells were coated with gal-streptavidin at 5 $\mu$g $ml^{-1}$ in 0.05 M carbonate buffer, pH 9.6 for 1 h and blocked with 3% BSA in PBS. Dilutions of mouse serum, from 1:100, and of a mouse monoclonal anti-streptavidin antibody standard (Monosan, Am Uden, The Netherlands) were added to the wells for 1 h, washed with PBS/Tween and water, and finally a 1:3000 dilution of peroxidase-linked sheep anti-mouse Ig (Amersham, Little Chalfont, UK) was added. The wells were washed and the assay was then developed with *o*-phenylenediamine dihydrochloride, blocked with 4 M HCl and the OD at 490 nm read. Anti-gal-streptavidin reactivity in each of the sera was related to the antibody titre of the control anti-streptavidin antibody standard for each assay to take into account any variation in absorbance readings between assays which would occur if the reaction was not stopped at exactly the same time each time the assay was carried out. Therefore, positive anti-gal-streptavidin response was calculated as a percentage of the control anti-streptavidin monoclonal antibody as follows:

$$\left(\frac{\text{titre of serum giving an OD reading of 0.3}}{\text{titre of control antibody giving an OD reading of 0.3}}\right) \times 100$$

An absorbance value of 0.3 was chosen because this fell within the linear range of the assay and generally included low positive antibody reactions.

### *Antibody preparation*

The antibody used in biodistribution studies was a murine monoclonal anti-CEA antibody, A5B7, which has previously been shown to localise to the human colon carcinoma xenograft LS174T (Pedley *et al.*, 1987). Biotinylation was carried out by adding caproylamidobiotin-NHS ester (Sigma) in dimethylsulphoxide (BDH) to A5B7 (1 mg $ml^{-1}$ in 0.1 M sodium bicarbonate, pH 8.5) at a 24:1 molar ratio. After incubation at room temperature for 4 h the antibody was dialysed against PBS. The resulting biotinylated A5B7 had 10 biotins per antibody molecule as assessed by the 4′-hydroxyazobenzene-2-carboxylic acid (HABA) dye assay (Pierce and Warriner) (Green, 1965). Iodination was carried out using the Iodo-gen method for 20 min to a specific activity of approximately 1 mCi $mg^{-1}$ protein and 94% of the [$^{125}$I]biotinylated A5B7 was found to bind CEA after labelling when tested by affinity chromatography on a CEA-Sepharose column. When A5B7 was iodinated to the same specificity, 98% of the labelled antibody was found to bind to the CEA-Sepharose column. Previous studies have shown no significant difference in the biodistribution and tumour localisation, to the LS174T xenograft, of [$^{125}$I]A5B7 before and after biotinylation (Marshall *et al.*, 1994), while in immunohistochemical studies on acetone-fixed cryostat or formalin-fixed paraffin sections of human tissues, biotinylated A5B7 gave an identical pattern of reactivity to the non-biotinylated A5B7 (data not shown).

### In vivo *biodistribution*

TO nude mice bearing the human colon carcinoma xenograft LS174T, established by subcutaneous passaging of the cell line LS174T (Tom *et al*., 1976), were injected via the tail vein with approximately 10 $\mu$g 10 $\mu$Ci$^{-1}$ [$^{125}$I]biotinylated antibody. Twenty-four hours after antibody injection test animals received an intraperitoneal injection of either 30 $\mu$g of gal-streptavidin or 30 $\mu$g PEG gal-streptavidin. Test animals were sacrificed and blood and tissues taken to be counted for radioactivity 1, 24 and 48 h after injection of gal-streptavidin or PEG gal-streptavidin (25, 48 and 72 h after antibody injection). Control animals, without administration of any clearing agent, were sacrificed at the same time points. The biodistribution data was calculated as percentage injected dose per gram of tissue (% ID/g) and are mean values of three or four mice per time point. In order to determine the size of circulating radioactive complexes in the mice, serum samples (taken 24 and 48 h after injection of the clearing agents) were applied to a 110 cm × 1 cm Sephacryl S300 gel filtration column and 1.3 ml fractions counted for $^{125}$I radioactivity in a multiwell gamma-counter.

### Results

### *Preparation of PEG-modified gal-streptavidin*

Figure 1 shows the effect of PEG modification on the apparent molecular weight of gal-streptavidin as assessed by gel filtration on a Sephacryl S300 column. 5 PEG gal-streptavidin, with a higher apparent molecular weight than unmodified gal-streptavidin, eluted earliest with a peak at fraction number 37 (48.1 ml), 2.5 PEG gal-streptavidin eluted slightly later with a peak at fraction 40 (52 ml), whereas gal-streptavidin had a peak at fraction number 46 (59.8 ml). The void volume of the column was found to be 37.7 ml (as assessed by the elution of blue dextran at fraction 29). The broad elution peaks of the PEG-modified gal-streptavidin samples indicated that different sized molecules were present in each preparation. This was to be expected, as the PEG reagent is comprised of different molecular weight molecules (average mol.wt. = 5000) and 2.5 PEG and 5 PEG refers only to an average number of PEG molecules conjugated to gal-streptavidin. If the apex of the peak is used to calculate the average molecular weight of the protein, then values of approximately 188 000 and 326 000 are obtained for 2.5 PEG and 5 PEG gal-streptavidin respectively, which are higher than the expected molecular weight of the proteins. This is thought to be due to the random coil structure of the PEG, which produces a much larger increase in the hydrodynamic radius of the protein than expected from the molecular mass alone (Delgado *et al*., 1992).

*In vitro* biotin binding of gal-streptavidin was impaired after PEG modification as shown by ELISA (Figure 2). However, this assay also indicates that the PEG-modified gal-streptavidin must still have at least two functional biotin-binding sites, since it was sandwiched between biotinylated antibody bound to the CEA wells and the biotin peroxidase used in the final stage of the assay.

### *Immunogenicity studies*

The effect of PEG modification on the immunogenicity of gal-streptavidin is shown in Figure 3. The anti-gal-streptavidin response in each mouse is expressed as a percentage of a positive control antibody standard. Figure 3a shows gal-streptavidin to be immunogenic in mice, with mice eliciting an anti-gal-streptavidin response in the range of 1.0–2.1% of the control 14 days after the primary injection, increasing to 7.5–91.7% and 24.4–156% of the control 7 and 14 days after the boost injection of gal-streptavidin (21 and 28 days after primary injection). Mice injected with 2.5 PEG gal-streptavidin (Figure 3b) showed a range in anti-gal-streptavidin response of 0–7.2% of the control antibody 14 days after primary immunisation, and although the anti-gal-streptavidin antibody titres increased after the boost injection, this was only to a maximum of 10.0% of the control antibody 21 days after primary injection (the remaining three mice had an anti-gal-streptavidin response of less than 5.0%), and to a maximum of 33.8% of the control antibody 28 days post primary injection (with the remaining three mice showing an anti-gal-streptavidin response of less than 11.0% of the control antibody). Increasing the degree of modification to 5 PEG molecules per gal-streptavidin dramatically reduced its immunogenicity in mice, with only two of the four mice showing very low levels of anti-gal-streptavidin antibodies after the two injections of 5 PEG gal-streptavidin (Figure 3c). Of these mice, one had an antibody titre of only 1.8% of the control antibody, 28 days after primary injection, and one mouse had a response that was lower than the usual limits of the assay, but was estimated to be less than 0.5% of the control antibody. The remaining two mice, in which no anti-gal-streptavidin response was detected, were injected 1 week later with unmodified gal-streptavidin, followed by a boost injection of gal-streptavidin 2 weeks later. No anti-gal-streptavidin response was detected, up to 28 days after the primary gal-streptavidin injection (14 days after the boost injection).

**Figure 1** Effect of PEG modification on the apparent molecular weight of gal-streptavidin as assessed by gel filtration on Sephacryl S300. ◆, gal-streptavidin; ●, 2.5 PEG gal-streptavidin; □, 5 PEG gal-streptavidin. Markers for the void volume, 669 kDa and 200 kDa were also applied to the column.

**Figure 2** Effect of PEG modification on the biotin binding of gal-streptavidin (◆), 2.5 PEG gal-streptavidin (○) and 5 PEG gal-streptavidin (■) assessed by ELISA.

*Effect of PEG modification on gal-streptavidin clearance of biotinylated A5B7*

*2.5 PEG-modified gal-streptavidin* When 2.5 PEG gal-streptavidin was administered 24 h after injection of

**Figure 3** Anti-gal-streptavidin response in individual mice after injection of gal-streptavidin or PEG-modified gal-streptavidin. Mice were injected with 30 μg of (**a**) gal-streptavidin, (**b**) 2.5 PEG gal-streptavidin or (**c**) 5 PEG gal-streptavidin on days 0 and 14, bled on days 7, 14, 21 and 28 and sera tested for anti-gal-streptavidin antibodies by ELISA. Results are expressed as a percentage of the anti-streptavidin control antibody.

**Figure 4** Biodistribution of [$^{125}$I]biotinylated A5B7 (**a**) 1 h, (**b**) 24 h and (**c**) 48 h after injection of gal-streptavidin (□) or 2.5 PEG gal-streptavidin (▨) clearing agents. Test animals were injected with the clearing agent 24 h after antibody injection and compared with animals injected with [$^{125}$I]biotinylated A5B7 alone (■). Vertical bars indicate s.d.

[$^{125}$I]biotinylated A5B7 no effect on the circulating [$^{125}$I]biotinylated A5B7 was observed 1 h after injection (Figure 4a). In contrast, administration of the unmodified gal-streptavidin clearing agent resulted, as expected, in a rapid decrease in blood radioactivity from 13.6% ID/g to 3.6% ID/g, with the gal-streptavidin-biotinylated antibody complexes being cleared through the liver.

Twenty-four hours after administration of 2.5 PEG gal-streptavidin (Figure 4b) the level of radioactivity in the blood was again similar to the control animals without any clearing agent. By this time point though, a reduction in tumour radioactivity from 24.4% ID/g to 14.3% ID/g was observed, thus resulting in a tumour to blood ratio of only $2.0 \pm 0.13$. The effect of administration of unmodified gal-streptavidin on the biodistribution of [$^{125}$I]biotinylated A5B7 is shown in comparison, and although a reduction in antibody localised at the tumour was also observed (from 24.4% ID/g to 12.5% ID/g), the increased blood clearance to only 0.8% ID/g resulted in an improved tumour to blood ratio of $16.7 \pm 5.3$.

Figure 4c shows that by 48 h after injection of 2.5 PEG gal-streptavidin a reduction in blood radioactivity was noted (from 7.6% ID/g to 2.4% ID/g), although this was not as great as that seen after administration of the unmodified gal-streptavidin (0.28% ID/g). A similar decrease in tumour radioactivity was also observed after administration of 2.5 PEG gal-streptavidin, therefore resulting in no improvement in the tumour to blood ratio ($2.5 \pm 1.2$ compared with $3.8 \pm 2.2$ in control animals without administration of clearing agent), whereas the unmodified gal-streptavidin gave an improved tumour to blood ratio of $29.7 \pm 14.4$ (range 16.9 to 48.8).

*5 PEG-modified gal-streptavidin* 5 PEG gal-streptavidin had no effect on the biodistribution of [$^{125}$I]biotinylated A5B7 1 h after administration, whereas unmodified gal-streptavidin reduced the level of circulating [$^{125}$I]biotinylated A5B7 3.3-fold from 15.5% ID/g in the controls to 4.7% ID/g (Figure 5a).

By 24 hours there was still little difference between the radioactivity levels in the circulation of control animals and those injected with 5 PEG gal-streptavidin clearing agent (Figure 5b), but tumour levels of the latter were reduced by half to 16.6% ID/g, resulting in a poor tumour to blood ratio of $1.5 \pm 0.8$ compared with $2.5 \pm 1.0$ in antibody alone control animals. Clearance with unmodified gal-streptavidin gave a corresponding tumour to blood ratio of $29.6 \pm 23.5$ (range from 9.9 to 63.2).

A similar pattern was again seen 48 h after administration of the clearing agents (Figure 5c), with a further reduction in

**Figure 5** Biodistribution of [$^{125}$I]biotinylated A5B7 (**a**) 1 h, (**b**) 24 h and (**c**) 48 h after injection of gal-streptavidin (□) or 5 PEG gal-streptavidin (▨) clearing agents. Test animals were injected with the clearing agent 24 h after antibody injection and compared with animals injected with [$^{125}$I]biotinylated A5B7 alone (■). Vertical bars indicate s.d.

**Figure 6** Analysis of serum by gel filtration on Sephacryl S300 24 h after administration of 2.5 PEG gal-streptavidin (○) or 48 h after administration of 5 PEG gal-streptavidin (■) (PEG gal-streptavidin was administered 24 h after [$^{125}$I]biotinylated A5B7 injection). [$^{125}$I]biotinylated A5B7 control was also applied to the column (◆).

tumour activity after injection of 5 PEG gal-streptavidin (to 9.1% ID/g), resulting in a tumour to blood ratio of only $1.4 \pm 0.2$ compared with $3.1 \pm 1.5$ for the antibody alone control animals and $48.1 \pm 16.1$ (range from 33.6 to 68.6) after clearance with unmodified gal-streptavidin. The blood clearance 24 and 48 h after injection of the unmodified gal-streptavidin was even greater than the corresponding groups previously presented in Figure 4, resulting in higher tumour to blood ratios. The reason for this further improvement in gal-streptavidin clearance in this series of data is not known.

*Gel filtration analysis of serum*

In order to ascertain whether the $^{125}I$ radioactivity remaining in the blood 24 and 48 h after administration of 2.5 or 5 PEG gal-streptavidin was circulating as free [$^{125}I$]biotinylated A5B7 or as a PEG gal-streptavidin–biotinylated antibody complex, mouse serum samples were applied to a Sephacryl S300 gel filtration column. Figure 6 shows that the $^{125}I$ radioactivity in the serum was eluted as a high molecular weight complex, at fraction 29 (equivalent to the void volume), with no free [$^{125}I$]biotinylated A5B7 being detectable.

### Discussion

Radioimmunotherapy is often limited by the persistence of radiolabelled antibody in the circulation, which causes bone marrow toxicity and limits the dose of radioactivity that can be safely administered to the patient. Various strategies to circumvent myelotoxicity have included using second antibody clearing agents to complex and clear the first, anti-tumour antibody (Begent *et al.*, 1982; Sharkey *et al.*, 1988; Pedley *et al.*, 1989), and extracorporeal immunoadsorption using either an anti-mouse column to reduce the level of circulating murine antibody (Lear *et al.*, 1991) or an avidin column to reduce the blood background levels of biotinylated antibodies (Norrgren *et al.*, 1993). Extracorporeal immunoadsorption has an advantage over other clearance systems of not causing accumulation of antibody complexes in the liver and spleen. Also Press *et al.* (1993) have reported impressive responses to high-dose radioimmunotherapy of B-cell lymphoma, with myelotoxic side-effects being minimised with autologous bone marrow support.

Other clearing and pretargeting strategies which have been investigated for reducing the dose of radioactivity to the blood involve the use of avidin or streptavidin which, being of non-human origin, invoke an immune response in humans. In one study 7/12 patients were reported by Paganelli *et al.* (1991*a*) to have developed anti-avidin antibodies after injection of 5 mg of avidin. Antibody therapy has frequently been hampered by the immunogenicity of murine monoclonal antibodies, although less immunogenic humanised antibodies are now becoming more widely available for clinical use. There is great interest in the development of a clearing agent that is also non-immunogenic and therefore an investigation to reduce the immunogenicity of our gal-streptavidin clearing agent (Marshall *et al.*, 1995) was undertaken.

Polyethylene glycol (PEG) is a linear, water-soluble, uncharged, flexible polymer that is available in various molecular weights and can be readily conjugated to proteins. PEG conjugation has been reported to abolish or reduce the immunogenicity of many proteins and was therefore an ideal candidate in an attempt to reduce the immunogenicity of our gal-streptavidin clearing agent.

The conjugation of PEG to gal-streptavidin was found to decrease its immunogenicity in mice (Figure 3). Anti-gal-streptavidin antibody titres after injection of 2.5 PEG gal-streptavidin were lower than those of control animals injected with unmodified gal-streptavidin. When the degree of PEG modification was increased to 5 PEG molecules per gal-streptavidin, only two out of four mice elicited an immune response, and in these mice the response was not only weaker, but was also later after primary injection of the protein. These findings are similar to those of Katre (1990) who noted that only three out of ten rabbits mounted an immune response to PEG-IL2, compared with ten out of ten immunised with unmodified interleukin 2 (IL-2), and did so later than those injected with IL-2. It was also noted that an increase in the degree of PEG modification further reduced the immunogenicity of the IL-2 protein in mice, as found with our PEG gal-streptavidin.

It has been reported that tolerance can be induced in mice after immunisation with PEG-conjugated proteins, depending on the degree of PEG modification and dose of PEG protein (Savoca *et al.*, 1984). Wilkinson *et al.* (1987*a*) found an approximately 80% reduction in the antibody response to human IgG if PEG-human IgG had been injected 6–43 days before, and it has also been reported that induced tolerance can be transferred to naive mice by T cells and T cell extracts (Wilkinson *et al.*, 1987*b*). It may be possible to induce tolerance to gal-streptavidin with PEG gal-streptavidin, as indicated by the two mice that failed to show any immune response to gal-streptavidin after prior injection of 5 PEG gal-streptavidin, although a full investigation with more animals is needed to confirm this finding.

Loss in bioactivity after PEG modification has been frequently noted for many proteins. Although increasing the number of PEG molecules per gal-streptavidin also reduced the immunogenicity, this increase in PEG conjugation also resulted in a significant decrease in biotin binding of the gal-streptavidin when tested by ELISA (Figure 2). Increasing the degree of PEG modification has been reported by others to result in a decrease in the bioactivity of the protein (Wieder *et al.*, 1979; Kitamura *et al.*, 1991; Tsutsumi *et al.*, 1995) and although early PEG modification methods were particularly harsh, protein inactivation has been reduced by using different conjugation methods (Abuchowski *et al.*, 1984). Several reasons for the reduction in bioactivity after PEG modification have been proposed, including conjugation of PEG to residues that are vital for the reactivity of the protein (Wieder *et al.*, 1979; Veronese, 1994). This is unlikely to be the reason for the loss in biotin binding after PEG conjugation to gal-streptavidin, as the PEG was linked via lysine residues that are not considered to be involved in the biotin binding process of streptavidin (Wilchek and Bayer, 1989). PEG modification is not thought to cause gross conformational changes as circular dichroism and nuclear magnetic resonance (NMR) spectroscopic studies by Suzuki *et al.* (1984) and Banci *et al.* (1990) did not show any significant differences between unmodified and PEG-modified proteins. It has been suggested that the decrease in bioactivity after PEG conjugation is due to interference with substrate approach to the active site. Yoshinaga *et al.* (1987) found that alkaline phosphatase conjugated with low molecular weight PEG had greater activity than when modified to the same degree of lysine substitution but with higher molecular weight PEG, indicating that the long chain of the polymer itself sterically hinders the substrate from binding to the active site.

Both forms of PEG gal-streptavidin were tested to assess whether their ability to complex and clear [$^{125}I$]biotinylated antibodies from the circulation had been altered. One hour after gal-streptavidin injection neither the 2.5 nor the 5 PEG gal-streptavidin had reduced the circulating radioactivity, whereas unmodified gal-streptavidin had reduced the blood radioactivity by approximately 3-fold. It has been suggested that PEG-modified proteins are slower in their diffusion from a subcutaneous injection site than unmodified proteins (Dreborg and Akerblom, 1990) and therefore the lack of blood clearance at this early time point was initially thought to be due to PEG gal-streptavidin taking longer to gain access to the blood stream from the peritoneal cavity.

The results obtained 24 h after PEG gal-streptavidin were rather surprising in that, although blood radioactivity levels were similar to the control animals without clearing agent, the tumour levels were reduced significantly (1.7-fold and 2-fold reduction after 2.5 and 5 PEG gal-streptavidin respectively). A similar finding was observed 48 h after administration of the 5 PEG molecule and, although blood radioactivity levels after injection of the 2.5 PEG gal-streptavidin were less than the control animals with [$^{125}$I]biotinylated A5B7 alone, the low levels of radioactivity in the tumour resulted in a tumour to blood ratio of only $2.5 \pm 1.2$ compared with a tumour to blood ratio of $29.7 \pm 14.4$ after administration of unmodified gal-streptavidin. The gel filtration of the serum at these later time points after PEG gal-streptavidin administration (Figure 6) indicated that the activity in the blood was circulating as a high molecular weight complex. Therefore, although the biotin binding of PEG gal-streptavidin was inhibited *in vitro*, the PEG modified gal-streptavidin was still able to bind to the [$^{125}$I]biotinylated A5B7 *in vivo* sufficiently to form large radiolabelled complexes (as shown by the shift in the peak of radioactivity from fraction 42, as expected for [$^{125}$I]biotinylated A5B7, to elution in the void volume of the column). PEG modified proteins are known to have an extended half-life *in vivo* (Kamisaki *et al.*, 1981; Tsutsumi *et al.*, 1995) irrespective of whether the usual pattern of clearance is via the reticuloendothelial system, is receptor-mediated carbohydrate recognition, or passive excretion (Beauchamp *et al.*, 1983). PEG is thought to act as a shield around the protein that protects it from elimination from the body, making clearance slower without changing the route of clearance, as shown by competition experiments of Beauchamp *et al.* (1983). Therefore, it appears that even though the [$^{125}$I]biotinylated antibody was part of a high molecular weight complex that would normally be cleared rapidly from the body via asialoglycoprotein receptors in the liver, the PEG component of the PEG gal-streptavidin-bound [$^{125}$I]biotinylated antibody prevented its rapid removal from the circulation, presumably by masking receptor recognition.

The radioactivity at the tumour site is thought to be maintained at a high level if the blood levels of the radiolabelled antibody are also high, due to the existence of an equilibrium between bound and non-bound, circulating antibody. Therefore, rapid removal of circulating antibody after administration of a clearing agent disrupts this equilibrium, resulting in reduced levels of antibody associated with the tumour (Sharkey *et al.*, 1988,1992). In this present study, it appears that the radiolabelled antibody was present in the circulation only as part of a high molecular weight complex, not as free [$^{125}$I]biotinylated antibody. Therefore, the antibody would be inhibited from binding at the tumour site. Any equilibrium between tumour-bound and circulating antibody would be disrupted and any antibody that dissociated from the tumour or was shed with the tumour antigen would not be replenished, therefore, although radioactivity was still elevated in the circulation after PEG gal-streptavidin, the high levels of radioactivity at the tumour site could not be maintained.

In conclusion, while the immunogenicity of a streptavidin clearing agent can be successfully reduced by conjugation with PEG, this advantage is compromised by the slow clearance of the subsequent PEG gal-streptavidin–biotinylated antibody complexes, which results in very limited reduction in the level of damaging radioactivity circulating in the blood, while also reducing tumour accumulation. PEG modification is very appealing for many non-human proteins used in the clinic, but the use of PEG for decreased immunogenicity of an antibody clearing agent is clearly limited. Preliminary data suggests that PEG gal-streptavidin could make the mice tolerant to the unmodified clearing agent. PEG proteins have previously been implicated as tolerogens in mice and, although this is an attractive use for PEG-modified gal-streptavidin, the ability of PEG-modified proteins to induce tolerance in man is yet to be tested. The gal-streptavidin used in this series of experiments had relatively low amounts of galactose compared with previous gal-streptavidin clearing agents that have been used. It may be possible that if the degree of galactosylation was increased, then faster clearance via the asialoglycoprotein receptor may still be possible even after PEG conjugation and this is under investigation.

**Acknowledgements**

A5B7 was kindly provided by Celltech Limited, Slough, UK. Immunhistochemical studies were carried out by GM Boxer in the Department of Clinical Oncology. This work was supported by the Cancer Research Campaign.

### References

ABUCHOWSKI A, McCOY JR, PALCZUK NC, VAN ES T AND DAVIS FF. (1977). Effect of covalent attachment of polyethylene glycol on immunogenicity and circulating life of bovine liver catalase. *J. Biol. Chem.*, **252**, 3582–3586.

ABUCHOWSKI A, KAZO G, VERHOEST JR CR, VAN ES T, KAFKEWITZ D, NUCCI ML, VIAU AT AND DAVIS FF. (1984). Cancer therapy with chemically modified enzymes. I. Antitumour properties of polyethylene glycol–asparaginase conjugates. *Cancer Biochem. Biophys.*, **7**, 175–186.

BANCI L, BERTINI I, CALICETI P, MONSU' SCOLARO L, SCHIAVON O AND VERONESE FM. (1990). Spectroscopic characterization of polyethyleneglycol-modified superoxide dismutase: $^1$H NMR studies on its $Cu_2Co_2$ derivative. *J. Inorg. Biochem.*, **39**, 149–159.

BEAUCHAMP CO, GONIAS SL, MENAPACE DP AND PIZZO SV. (1983). A new procedure for the synthesis of polyethylene glycol-protein adducts; effects on function, receptor recognition, and clearance of superoxide dismutase, lactoferrin, and $\alpha_2$-macroglobulin. *Anal. Biochem.*, **131**, 25–33.

BEGENT RHJ, KEEP PA, GREEN AJ, SEARLE F, BAGSHAWE KD, JEWKES RF, JONES BE, BARRATT GM AND RYMAN BE. (1982). Liposomally entrapped second antibody improves tumour imaging with radiolabelled (first) antitumour antibody. *Lancet*, **2**, 739–742.

DELGADO C, FRANCIS GE AND FISHER D. (1992). The uses and properties of PEG-linked proteins. *Crit. Rev. Therap. Drug Carrier Syst.*, **9**, 249–304.

DREBORG S AND AKERBLOM EB. (1990). Immunotherapy with monomethoxypolyethylene glycol modified allergens. *Crit. Rev. Therap. Drug Carrier Syst.*, **6**, 315–365.

DUBOIS M, GILLES KA, HAMILTON JK, REBERS PA AND SMITH F. (1956). Colorimetric method for determination of sugars and related substances. *Anal. Chem.*, **28**, 350–356.

FRAKER PJ AND SPECK JC. (1978). Protein and cell membrane iodinations with a sparingly soluble chloroamide, 1,3,4,6-tetrachloro-3a,6a-diphenylglycoluril. *Biochem. Biophys. Res. Commun.*, **80**, 849–857.

GREEN NM. (1965). A spectrophotometric assay for avidin and biotin based on binding of dyes by avidin. *Biochem. J.*, **94**, 23c–24c.

HALE G, DYER MJS, CLARK MR, PHILLIPS JM, MARCUS R, REICHMANN L, WINTER G AND WALDMANN H. (1988). Remission induction in non-Hodgkin lymphoma with reshaped human monoclonal antibody CAMPATH-1H. *Lancet*, **2**, 1394–1399.

HIRD V, VERHOEYEN M, BADLEY RA, PRICE D, SNOOK D, KOSMAS C, GOODEN C, BAMIAS A, MEARES C, LAVENDER JP AND EPENETOS AA. (1991). Tumour localisation with a radioactively labelled reshaped human monoclonal antibody. *Br. J. Cancer*, **64**, 911–914.

KAMISAKI Y, WADA H, YAGURA T, MATSUSHIMA A AND INADA Y. (1981). Reduction in immunogenicity and clearance rate of *Escherichia coli* L-asparaginase by modification with monomethoxypolyethylene glycol. *J. Pharmacol. Exp. Ther.*, **216**, 410–414.

KATRE NV. (1990). Immunogenicity of recombinant IL-2 modified by covalent attachment of polyethylene glycol. *Immunol. J.*, **144**, 209–213.

KHAWLI LA, ALAUDDIN MM, MILLER GK AND EPSTEIN AL. (1993). Improved immunotargeting of tumors with biotinylated monoclonal antibodies and radiolabeled streptavidin. *Antib. Immunoconj. Radiopharm.*, **6**, 13–27.

KITAMURA K, TAKAHASHI T, YAMAGUCHI T, NOGUCHI A, NOGUCHI A, TAKASHINA K, TSURUMI H, INAGAKE M, TOYOKUNI T AND HAKOMORI S. (1991). Chemical engineering of the monoclonal antibody A7 by polyethylene glycol for targeting cancer chemotherapy. *Cancer Res.*, **51**, 4310–4315.

LEAR JL, KASLIWAL RK, FEYERABEND AJ, PRATT JP, BUNN PA, DIENHART DG, GONZALEZ R, JOHNSON TK, BLOEDOW DC, MADDOCK SW AND GLENN SD. (1991). Improved tumor imaging with radiolabeled monoclonal antibodies by plasma clearance of unbound antibody with anti-antibody column. *Radiology*, **179**, 509–512.

LEDERMANN JA, BEGENT RHJ, BAGSHAWE KD, RIGGS SJ, SEARLE F, GLASER MG, GREEN AJ AND DALE RG. (1988). Repeated antitumour antibody therapy in man with suppression of the host response by Cyclosporin A. *Br. J. Cancer*, **58**, 654–657.

LEE WY AND SEHON AH. (1977). Abrogation of reaginic antibodies with modified allergens. *Nature*, **267**, 618–619.

LoBUGLIO AF, WHEELER RH, TRANG J, HAYNES A, ROGERS K, HARVEY EB, SUN L, GHRAYEB J AND KHAZAELI MB. (1989). Mouse/human chimeric monoclonal antibody in man: kinetics and immune response. *Proc. Natl Acad. Sci. USA.*, **86**, 4220–4224.

LYONS A, KING DJ, OWENS RJ, YARRANTON GT, MILLICAN A, WHITTLE NR AND ADAIR JR. (1990). Site-specific attachment to recombinant antibodies via introduced surface cysteine residues. *Protein Eng.*, **3**, 703–708.

MARSHALL D, PEDLEY RB, BODEN JA, BODEN R AND BEGENT RHJ. (1994). Clearance of circulating radio-antibodies using streptavidin or second antibodies in a xenograft model. *Br. J. Cancer*, **69**, 502–507.

MARSHALL D, PEDLEY RB, MELTON RG, BODEN JA, BODEN R AND BEGENT RHJ. (1995). Galactosylated streptavidin for improved clearance of biotinylated intact and $F(ab')_2$ fragments of an anti-tumour antibody. *Br. J. Cancer*, **71**, 18–24.

NORRGREN K, STRAND S-E, NILSSON R, LINDGREN L AND SJOGREN H-O. (1993). A general, extracorporeal immunosorption method to increase the tumor to normal tissue ratio in radioimmunoimaging and radioimmunotherapy. *J. Nucl. Med.*, **34**, 448–454.

PAGANELLI G, STELLA M, DE NARDI P, MAGNANI P, ZITO F, SICCARDI AG, DI CARLO V AND FAZIO F. (1990*a*). A new method for faster blood clearance in radioimmuno-guided surgery. *J. Nucl. Med. Allied Sci.*, **35**, 88–89.

PAGANELLI G, PERVEZ S, SICCARDI AG, ROWLINSON G, DELEIDE G, CHIOLERIO F, MALCOVATI M, SCASSELLATI GG AND EPENETOS AA. (1990*b*). Intraperitoneal radio-localistion of tumours pre-targeted by biotinylated monoclonal antibodies. *Int. J. Cancer*, **45**, 1184–1189.

PAGANELLI G, MAGNANI P, ZITO F, VILLA E, SUDATI F, LOPALCO L, ROSSETI L, MALCOVATI M, CHIOLERIO F, SECCAMANI E, SICCARDI AG AND FAZIO F. (1991*a*). Three-step monoclonal antibody tumor targeting in carcinoembryonic antigen-positive patients. *Cancer Res.*, **51**, 5960–5966.

PAGANELLI G, MALCOVATI M AND FAZIO F. (1991*b*). Monoclonal antibody pretargeting techniques for tumour localization: the avidin-biotin system. *Nucl. Med. Commun.*, **12**, 211–234.

PAGANELLI G, BELLONI C, MAGNANI P, ZITO F, PASINI A, SASSI I, MERONI M, MARIANI M, VIGNALI M, SICCARDI AG AND FAZIO F. (1992). Two-step tumour targeting in ovarian cancer patients using biotinylated monoclonal antibodies and radioactive streptavidin. *Eur. J. Nucl. Med.*, **19**, 322–329.

PEDLEY RB, BODEN J, KEEP PA, HARWOOD PJ, GREEN AJ AND ROGERS GT. (1987). Relationship between tumour size and uptake of radiolabelled anti-CEA in a colonic tumour xenograft. *Eur. J. Nucl. Med.*, **13**, 197–202.

PEDLEY RB, DALE R, BODEN JA, BEGENT RHJ, KEEP PA AND GREEN AJ. (1989). The effect of second antibody clearance on the distribution and dosimetry of radiolabelled anti-CEA antibody in a human colonic tumour xenograft model. *Int. J. Cancer*, **43**, 713–718.

PEDLEY RB, BODEN J, BODEN R, BEGENT RHJ, TURNER A, HAINES AMR AND KING DJ. (1994). The potential for enhanced tumour localistion by poly(ethylene glycol) modification of anti-CEA antibody. *Br. J. Cancer*, **70**, 1126–1130.

PRESS OW, EARY JF, APPELBAUM FR, MARTIN PJ, BADGER CC, NELP WB, GLENN S, BUTCHKO G, FISHER D, PORTER B, MATTHEWS DC, FISHER LD AND BERNSTEIN ID. (1993). Radiolabeled-antibody therapy of B-cell lymphoma with autologous bone marrow support. *N. Engl. J. Med.*, **329**, 1219–1224.

SAGA T, WEINSTEIN JN, JEONG JM, HEYA T, LEE JT, LE N, PAIK CH, SUNG C AND NEUMANN RD. (1994). Two-step pretargeting of experimental lung metastases with biotinylated antibody and radiolabeled streptavidin. *Cancer Res.*, **54**, 2160–2165.

SAVOCA KV, DAVIS FF AND PALCZUK NC. (1984). Induction of tolerance in mice by uricase and monomethoxypolyethylene glycol-modified uricase. *Int. Arch. Allergy Appl. Immun.*, **75**, 58–67.

SHARKEY RM, MABUS J AND GOLDENBERG DM. (1988). Factors influencing anti-antibody enhancement of tumor targeting with antibodies in hamsters with human colonic tumor xenografts. *Cancer Res.*, **48**, 2005–2009.

SHARKEY RM, BOERMAN OC, NATALE A, PAWLYK D, MONESTIER M, LOSMAN MJ AND GOLDENBERG DM. (1992). Enhanced clearance of radiolabeled murine monoclonal antibody by a syngeneic anti-idiotype antibody in tumor-bearing nude mice. *Int. J. Cancer*, **51**, 266–273.

SINITSYN VV, MAMONTOVA AG, CHECKNEVA YY, SHNYRA AA AND DOMOGATSKY SP. (1989). Rapid blood clearance of biotinylated IgG after infusion of avidin. *J. Nucl. Med.*, **30**, 66–69.

SUZUKI T, KANBARA N, TOMONO T, HAYASHI N AND SHINOHARA I. (1984). Physicochemical and biological properties of poly(ethylene glycol)-coupled immunoglobulin G. *Biochim. Biophys. Acta*, **788**, 248–255.

TOM BH, RUTZKY LP, JAKSTYS MM, OYASU R, KAYE CI AND KAHAN BD. (1976). Human colonic adenocarcinoma cells. *In Vitro*, **12**, 180–191.

TSUTSUMI Y, KIHIRA T, TSUNODA S, KANAMORI T, NAKAGAWA S AND MAYUMI T. (1995). Molecular design of hybrid tumour necrosis factor alpha with polyethylene glycol increases its anti-tumour potency. *Br. J. Cancer*, **71**, 963–968.

TURNER A, KING DJ, FARNSWORTH APH, RHIND SK, PEDLEY RB, BODEN J, BODEN R, MILLICAN TA, MILLAR K, BOYCE B, BEELEY NRA, EATON MAW AND PARKER D. (1994). Comparative biodistributions of indium-111-labelled macrocycle chimeric B72.3 antibody conjugates in tumour-bearing mice. *Br. J. Cancer*, **70**, 35–41.

VERONESE FM. (1994). Enzyme surface modification by polymers for improved delivery. *J. Control. Release*, **29**, 171–176.

WIEDER KJ, PALCZUK NC, VAN ES T AND DAVIS FF. (1979). Some properties of polyethylene glycol:phenylalanine ammonia-lyase adducts. *J. Biol. Chem.*, **254**, 12579–12587.

WILCHEK M AND BAYER EA. (1989). Avidin-biotin technology ten years on: has it lived up to its expectations? *Trends Biol. Sci.*, **14**, 408–413.

WILKINSON I, JACKSON C-JC, LANG GM, HOLFORD-STREVENS V AND SEHON AH. (1987*a*). Tolerogenic polyethylene glycol derivatives of xenogeneic monoclonal immunoglobulins. *Immunol. Lett.*, **15**, 17–22.

WILKINSON I, JACKSON C-JC, LANG GM, HOLFORD-STREVENS V AND SEHON AH. (1987*b*). Tolerance induction in mice by conjugates of monoclonal immunoglobulins and monomethoxypolyethylene glycol. Transfer of tolerance by T cells and by T cell extracts. *J. Immunol.*, **139**, 326–331.

YOSHINAGA K, SHAFER SG AND HARRIS JM. (1987). Effects of polyethylene glycol substitution on enzyme activity. *J. Bioact. Compat. Polym.*, **2**, 49–56.